# Effect of transcranial magnetic stimulation on the limb function of stroke patients: a systematic review and meta-analysis

**DOI:** 10.3389/fneur.2026.1833450

**Published:** 2026-07-03

**Authors:** Lisha Xie, Nangen Song, Cui Huang, Yong Fan, Youjia Mao, Zhicheng Zhu

**Affiliations:** School of Physical Education, Xinyu University, Xinyu, China

**Keywords:** limb function, meta-analysis, review, stroke patients, transcranial magnetic stimulation

## Abstract

**Objective:**

This study aims to evaluate the effects of transcranial magnetic stimulation (TMS) on Fugl-Meyer Assessment for Upper Limb (FMA-UL), Fugl-Meyer Assessment for Lower Limb (FMA-LL), Modified Ashworth Scale (MAS), Box and Block Test (BBT), Action Research Arm Test (ARAT), and Wolf Motor Function Test (WMFT) in stroke patients.

**Data sources:**

A systematic search of PubMed, Web of Science, Embase, and Ovid databases was conducted to identify relevant studies.

**Methods:**

Only randomized controlled trials (RCTs) investigating TMS interventions in stroke patients were included. Data analysis was performed using Stata 18.0, incorporating subgroup analysis, sensitivity analysis, and bias assessments.

**Results:**

A total of 21 documents were included. The meta-analysis revealed that TMS significantly improved ARAT (MD = 8.01, 95% CI: 0.53–15.50, *P* = 0.04) and BBT (MD = 5.36, 95% CI: 0.16–10.56, *P* = 0.04). However, no significant effects were observed for FMA-UL, FMA-LL, MAS, and WMFT (*P* > 0.88). The subgroup analysis shows that there is a significant difference in the effects of different intervention cycles on FMA-UL in stroke patients. The intervention cycle has no effect on FMA (*P* > 0.05), but weeks> 2 improves FMA (MD = 4.44, 95% CI: 0.83–8.06, *P* = 0.016).

**Conclusion:**

TMS has a positive effect on the limb function of stroke patients, especially the flexibility and motion function of the upper limbs. However, due to the limited sample size in this study, further large-scale RCTs are necessary to confirm these findings.

**Systematic review registration:**

https://www.crd.york.ac.uk/PROSPERO/myprospero, identifier: CRD42024618509.

## 1 Introduction

Stroke is the second leading cause of death globally and the primary cause of disability (1, 2). According to the Global Burden of Disease Report, approximately 101 million individuals were living with stroke worldwide in 2019, with 12.2 million new cases reported that year. Stroke accounted for 6.55 million deaths, representing 11.6% of total global mortality. And contributed to 143 million disability-adjusted life years (DALYs), equivalent to 5.7% of the total DALYs (2).

Although stroke patients often receive timely treatment, many survivors still experience varying degrees of dysfunction (3, 4). For instance, hemiplegia, a common post-stroke symptom, results in weakened or lost motor control, preventing free limb movement and significantly impairing daily activities (5). Limited limb mobility, particularly in the upper limbs, is another frequent challenge, making movement increasingly difficult for many patients (6). Fortunately, accumulating evidence suggests that transcranial magnetic stimulation (TMS) may confer therapeutic benefits for post-stroke hemiparesis by facilitating neuroplastic reorganization, modulating neural network excitability, and restoring cortical excitability balance (7–9). This potential has been further supported by several recent meta-analyses. For instance, Li et al. (10) reported that both excitatory and inhibitory repetitive TMS (rTMS) were associated with significant improvements in upper-limb motor performance among stroke survivors. Similarly, the network meta-analysis conducted by Keesukphan et al. (11) indicated that TMS, whether delivered as a stand-alone intervention or combined with other neuromodulatory approaches, produced beneficial effects on upper-limb function. In addition, Shim et al. (12) demonstrated that TMS could improve upper-limb function and manual dexterity, with subsequent gains in activities of daily living and health-related quality of life. Consistent with these findings, further evidence has also shown that TMS contributes to overall motor recovery after stroke (13).

Despite the promising findings, some controversies remain in this field. For instance, studies by Hosomi et al. (14) and Guan et al. (15) demonstrated that TMS significantly improves upper limb function in stroke patients, whereas Sharma et al. (16) and Meng et al. (17) reported no significant improvement. Additionally, the effects of different TMS frequencies are debated. For example, Zhang et al. (18) found that low-frequency TMS significantly enhances upper limb function, while Wang, Ray-Yau et al. (19) and others showed that high-frequency TMS produces similar benefits. Moreover, while Xi et al. (20) investigated the effects of repetitive TMS combined with task-oriented training on upper limb function in stroke patients, the impact of TMS on lower limb function remains understudied.

Given the inconsistencies across previous studies and the remaining gaps in the current evidence base, a comprehensive synthesis of the available literature is warranted. Such an analysis may help clarify the effects of TMS on different dimensions of post-stroke limb function and determine whether specific intervention characteristics influence treatment outcomes. Accordingly, this study aimed to systematically evaluate the efficacy of TMS in improving limb function among patients with stroke, with outcomes assessed using the Fugl-Meyer Assessment (FMA), Modified Ashworth Scale (MAS), Box and Block Test (BBT), Action Research Arm Test (ARAT), and Wolf Motor Function Test (WMFT). Furthermore, we explored whether the effects of TMS differed according to intervention modality, treatment duration, and stimulation frequency. We hypothesized that TMS would be beneficial for post-stroke limb functional recovery, although the magnitude of its effects may depend on stimulation parameters and intervention-related factors.

## 2 Method

### 2.1 Literature search

This study adhered to the Preferred Reporting Items for Systematic Reviews and Meta-Analysis (PRISMA) guidelines and the study protocol was registered with PROSPERO (CRD42024618509). A systematic literature search was performed in PubMed, Web of Science, Embase, and Ovid from database inception to October 13, 2024, to retrieve randomized controlled trials evaluating the effects of transcranial magnetic stimulation on limb function in individuals with stroke. The search strategy incorporated terms related to “transcranial magnetic stimulation,” “stroke,” and “randomized controlled trial,” which were combined using appropriate Boolean operators, including AND and OR. The full PubMed search strategy, including field tags, MeSH terms, and detailed search syntax, is provided in Table 1. To maximize the comprehensiveness of the search, reference lists of relevant reviews, systematic reviews, and meta-analyses were also manually screened for additional eligible studies.

**Table 1:** PubMed 474.

Search number	Query	Results
1	“Transcranial Magnetic Stimulation”[Mesh]	18,076
2	Magnetic Stimulations, Transcranial[Title/Abstract] OR Magnetic Stimulation, Transcranial[Title/Abstract] OR Stimulations, Transcranial Magnetic[Title/Abstract] OR Stimulation, Transcranial Magnetic[Title/Abstract] OR Transcranial Magnetic Stimulations[Title/Abstract] OR Transcranial Magnetic Stimulation, Paired Pulse[Title/Abstract] OR Transcranial Magnetic Stimulation, Repetitive[Title/Abstract] OR Transcranial Magnetic Stimulation, Single Pulse ‘Magnetic Stimulations, Transcranial'[Title/Abstract] OR ‘Magnetic Stimulation, Transcranial'[Title/Abstract] OR ‘Stimulations, Transcranial Magnetic'[Title/Abstract] OR ‘Stimulation, Transcranial Magnetic'[Title/Abstract] OR ‘Transcranial Magnetic Stimulations'[Title/Abstract] OR ‘Transcranial Magnetic Stimulation, Paired Pulse'[Title/Abstract] OR ‘Transcranial Magnetic Stimulation, Repetitive'[Title/Abstract] OR ‘Transcranial Magnetic Stimulation, Single Pulse'[Title/Abstract]	25,847
3	“Stroke”[Mesh]	199,916
4	Strokes[Title/Abstract] OR Cerebrovascular Accident[Title/Abstract] OR Cerebrovascular Accidents[Title/Abstract] OR Cerebral Stroke[Title/Abstract] OR Cerebral Strokes[Title/Abstract] OR Stroke, Cerebral[Title/Abstract] OR Strokes, Cerebral[Title/Abstract] OR Cerebrovascular Apoplexy[Title/Abstract] OR Apoplexy, Cerebrovascular[Title/Abstract] OR Vascular Accident, Brain[Title/Abstract] OR Brain Vascular Accident[Title/Abstract] OR Brain Vascular Accidents[Title/Abstract] OR Vascular Accidents, Brain[Title/Abstract] OR Cerebrovascular Stroke[Title/Abstract] OR Cerebrovascular Strokes[Title/Abstract] OR Stroke, Cerebrovascular[Title/Abstract] OR Strokes, Cerebrovascular[Title/Abstract] OR Apoplexy[Title/Abstract] OR CVA (Cerebrovascular Accident[Title/Abstract]) OR CVAs (Cerebrovascular Accident[Title/Abstract]) OR Stroke, Acute[Title/Abstract] OR Acute Stroke[Title/Abstract] OR Acute Strokes[Title/Abstract] OR Strokes, Acute[Title/Abstract] OR Cerebrovascular Accident, Acute[Title/Abstract] OR Acute Cerebrovascular Accident[Title/Abstract] OR Acute Cerebrovascular Accidents[Title/Abstract] OR Cerebrovascular Accidents, Acute[Title/Abstract]	29,281
5	“Randomized Controlled Trial” [Publication Type] OR “Randomized Controlled Trials as Topic”[Mesh] OR “Controlled Clinical Trial” [Publication Type]	941,724
6	#1 OR #2	28,129
7	#3 OR #4	211,819
8	#6 AND #7	1,832
9	#5 AND #8	474

### 2.2 Inclusion criteria

We included studies involving stroke patients who received TMS treatment. The research design was limited to randomized controlled trials. The experimental group underwent repetitive transcranial magnetic stimulation (rTMS) or intermittent theta burst stimulation (iTBS). The primary outcome measure was the Fugl-Meyer Assessment of Upper Limb (FMA-UL), while secondary outcome measures included the Fugl-Meyer Assessment of Lower Limb (FMA-LL), Motor Assessment Scale (MAS), Action Research Arm Test (ARAT), Box and Block Test (BBT), and Wolf Motor Function Test (WMFT).

### 2.3 Exclusion criteria

We excluded studies that involved non-stroke patients or patients with depression. If the experimental group employed electrical stimulation methods other than TMS, such as transcranial direct current stimulation or pharyngeal electrical stimulation, or if the control group did not use sham stimulation. We also excluded studies where the primary outcomes were serum brain-derived neurotrophic factor (BDNF) concentration, mortality, or disability rates, as well as non-randomized controlled trials such as cohort studies and observational studies. In addition, studies lacking sufficient information to calculate effect sizes, for which attempts to contact the corresponding authors via email were unsuccessful were excluded.

### 2.4 Data extraction

Two independent reviewers extracted the following information from each study: first author, publication year, sample size, participants' age and gender, intervention method, cycles, frequency, and intensity. Any discrepancies were resolved through discussion with a third reviewer until consensus was reached.

### 2.5 Risk of bias assessment

The systematic evaluation manual recommended by the Cochrane Collaboration Network assesses the following biases: random sequence generation, allocation concealment, blinding of subjects and experimenters, blinding of outcome assessors, completeness of outcome data, and selective reporting. Each bias was classified as high risk, low risk, or unclear risk.

### 2.6 Data analysis

Data were analyzed using Stata 18.0. The overall effect size was calculated using the mean difference (MD) and 95% confidence interval (CI); Heterogeneity was assessed using *P*-values and the I^2^ statistic. When I^2^ <50% and P ≥ 0.1, heterogeneity was considered low, and a fixed-effect model was used to calculate the combined effect. When I^2^ > 50% and *P* < 0.1, heterogeneity was considered high, and a random-effects model was used to calculate the combined effect. In addition, to further explore the optimal effects on indicators related to limb function in stroke patients, we conducted subgroup analysis based on intervention method (rTMS and iTBS), cycle (weeks ≤ 2 and weeks >2), and frequency (low frequency ≤ 1Hz and high frequency >1Hz). Sensitivity analysis and publication bias tests were also performed using Stata 18.0. A *P*-value ≤ 0.05 was considered statistically significant.

## 3 Results

### 3.1 Literature search results

**Figure 1:**
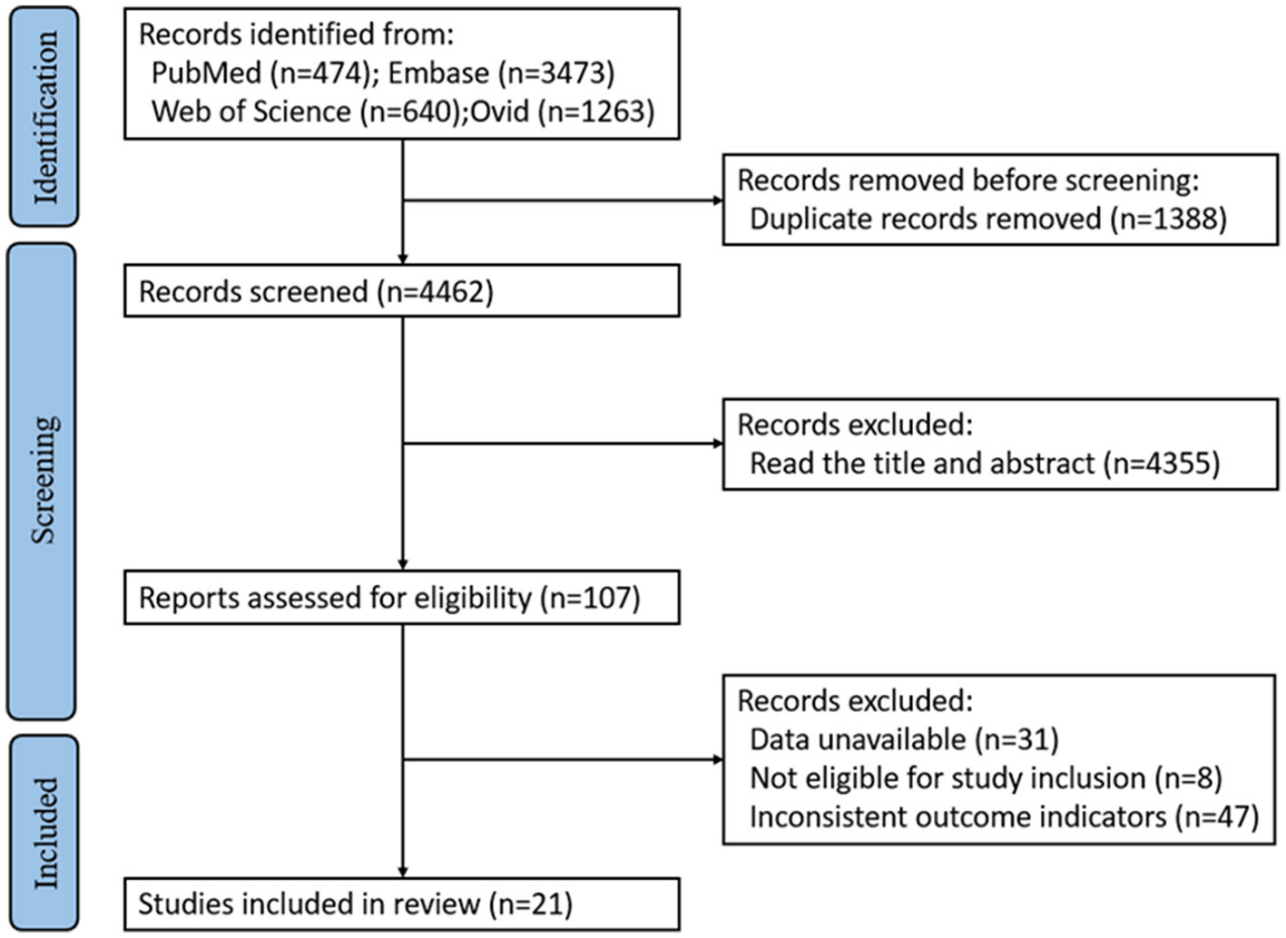
PRISMA flowchart.

A total of 5,850 articles were retrieved from the PubMed, Web of Science, Embase, and Ovid databases. After removing 1,388 duplicates using EndNote, 4,462 articles remained. Through screening of titles and abstracts, 4,355 articles were excluded, leaving 107 articles for full-text review. Of these, 86 articles were excluded: data could not be extracted from 31 articles, 8 articles did not meet the inclusion criteria, and 47 articles did not meet the outcome indicators. Ultimately, 21 articles were included in the meta-analysis (Figure 1).

### 3.2 Characteristics of the literature

In the 21 studies, the experimental groups in all studies described the intervention methods, including both rTMS and iTBS types. Sixteen studies used rTMS (12–14, 16, 17, 19, 21–30), and 5 studies used iTBS (15, 31–34). All control groups received sham stimulation. Additionally, 2 studies incorporated cycling training alongside iTBS (15, 31), with the control groups also receiving cycling training (Table 2).

**Table 2:** Basic information on the included literature.

Author	Year	*N* (E/C)	Type of intervention		Intervention period	Total sessions	Pulse number	Frequency	Motion threshold	Outcome
			E	C						
Mirowska-Guzel et al. (21)	2013	46 (23/23)	rTMS	Sham stimulation	3 weeks	15	1,800	1 HZ	90% RTM	FMA-UL
Chen et al. (31)	2021	23 (12/11)	iTBS+VCT	Sham iTBS+VCT	3 weeks	15	1,200	50 HZ	80% AMT	FMA-UL, MAS, ARAT, BBT
Higgins et al. (22)	2013	9 (4/5)	rTMS	Sham rTMS	4 weeks	8	1,200	1 HZ	110% RTM	BBT, WMFT
Hosomi et al. (14)	2016	39 (18/21)	rTMS	Sham rTMS	10 days	80	500	5 HZ	90% RTM	FMA-UL
Zheng et al. (23)	2015	112 (58/54)	rTMS+VR training	Sham rTMS+ VR training	4 weeks	24	1,800	1 HZ	90% RTM	WMFT
Lüdemann-Podubecká et al. (24)	2015	33 (17/16)	rTMS	Sham rTMS	3 weeks	15	900	1 HZ	100% RTM	WMFT
Guan et al. (15)	2017	42 (21/21)	iTBS+VCT	Sham iTBS+VCT	10 days	/	500	5 HZ	120% RTM	FMA-UL, FMA-LL
Shim et al. (12)	2023	30 (14/16)	rTMS+ ML	Sham rTMS + ML	4 weeks	200	200	10 HZ	80% RTM	FMA-UL, BBT
Kim et al. (32)	2015	15 (7/8)	iTBS	Sham iTBS	/	30	600	5 HZ	80% AMT	MAS
Barros Galvão et al. (25)	2014	20 (10/10)	rTMS+ PT	Sham stimulation + PT	4 weeks	10	1,500	1 HZ	90% RTM	FMA-UL, MAS
Sharma et al. (16)	2020	96 (47/49)	rTMS	Sham rTMS	2 weeks	10	750	1 HZ	110% RTM	FMA-UL, FMA-LL
Wang et al. (19)	2018	14 (8/6)	rTMS	Sham rTMS	3 weeks	/	900	5 HZ	90% RTM	FMA-LL
Chen et al. (33)	2013	22 (11/11)	iTBS	Sham iTBS	2 weeks	10	600	5 HZ	80% AMT	FMA-UL, MAS, ARAT, BBT
Luk et al. (26)	2022	24 (12/12)	rTMS	Sham rTMS	10 weeks	10	1,200	1 HZ	90% RTM	FMA-UL, ARAT
Lee et al. (27)	2024	20 (10/10)	rTMS	Sham rTMS	2 weeks	140	1,000	10 HZ	90% RTM	FMA-UL, BBT
Huang et al. (28)	2018	38 (18/20)	rTMS	Sham rTMS	3 weeks	15	900	1 HZ	120% AMT	FMA-LL
Malcolm et al. (13)	2007	19 (9/10)	rTMS	Sham rTMS	2 weeks	/	2,000	20 HZ	90% RTM	WMFT, BBT
Wang et al. (29)	2012	24 (12/12)	rTMS	Sham rTMS	2 weeks	/	600	1 HZ	90% RTM	FMA-LL
Meng et al. (17)	2024	34 (18/16)	rTMS	Sham rTMS	2 weeks	10	801	6 HZ	80% RTM	FMA-UL
Chang et al. (34)	2020	28 (14/14)	iTBS+rPMS	Sham rPMS+iTBS	2 weeks	/	600	5 HZ	70% RTM/80% AMT	FMA-UL, ARAT
Gottlieb et al. (30)	2021	28 (14/14)	rTMS	Sham rTMS	2 weeks	10	1,200	1 HZ	100% RTM	FMA-UL, MAS

### 3.3 Evaluation of risk bias

In the 21 studies, 16 studies clearly explained the randomization methods (14–17, 21, 23–31, 33, 34), while 5 studies did not explicitly describe the randomization methods (12, 13, 19, 22, 32); Only 6 studies mentioned whether group allocation was concealed (15–17, 24, 26, 29); Seventeen studies used a double-blind method (12, 14–17, 19, 21, 23–26, 28–30, 32–34), while the remaining studies did not. All predetermined outcome indicators were reported (Table 3).

**Table 3:** Risk of bias evaluation of included studies.

Author	Year	Random sequence generation	Allocation concealment	Blind		Incomplete outcome data	Selective reporting	Other bias
				Participants and personnel	Outcome assessment			
Mirowska-Guzel et al. (21)	2013	Low risk	Unclear risk	Low risk	Low risk	Unclear risk	Unclear risk	Unclear risk
Chen et al. (31)	2021	Low risk	Unclear risk	Unclear risk	Low risk	Low risk	Unclear risk	Unclear risk
Higgins et al. (22)	2013	Unclear risk	Unclear risk	Low risk	Unclear risk	Low risk	Unclear risk	Unclear risk
Hosomi et al. (14)	2016	Low risk	Unclear risk	Low risk	Low risk	Unclear risk	Unclear risk	Unclear risk
Zheng et al. (23)	2015	Low risk	Unclear risk	Low risk	Low risk	Unclear risk	Unclear risk	Unclear risk
Lüdemann-Podubecká et al. (24)	2015	Low risk	Low risk	Low risk	Low risk	Unclear risk	Unclear risk	Unclear risk
Guan et al. (15)	2017	Low risk	Low risk	Low risk	Low risk	Unclear risk	Unclear risk	Unclear risk
Shim et al. (12)	2023	Unclear risk	Unclear risk	Low risk	Low risk	Unclear risk	Unclear risk	Unclear risk
Kim et al. (32)	2015	Unclear risk	Unclear risk	Low risk	Low risk	Unclear risk	Unclear risk	Unclear risk
Barros Galvão et al. (25)	2014	Low risk	Unclear risk	Low risk	Low risk	Unclear risk	Unclear risk	Unclear risk
Sharma et al. (16)	2020	Low risk	Low risk	Low risk	Low risk	Unclear risk	Unclear risk	Unclear risk
Wang et al. (19)	2018	Unclear risk	Unclear risk	Low risk	Low risk	Unclear risk	Unclear risk	Unclear risk
Chen et al. (33)	2013	Low risk	Unclear risk	Low risk	Low risk	Unclear risk	Unclear risk	Unclear risk
Luk et al. (26)	2022	Low risk	Low risk	Low risk	Low risk	Unclear risk	Unclear risk	Unclear risk
Lee et al. (27)	2024	Low risk	Unclear risk	Unclear risk	Low risk	Unclear risk	Unclear risk	Low risk
Huang et al. (28)	2018	Low risk	Unclear risk	Low risk	Low risk	Unclear risk	Unclear risk	Unclear risk
Malcolm et al. (13)	2007	Unclear risk	Unclear risk	Unclear risk	Unclear risk	Low risk	Unclear risk	Unclear risk
Wang et al. (29)	2012	Low risk	Low risk	Low risk	Low risk	Unclear risk	Unclear risk	Unclear risk
Meng et al. (17)	2024	Low risk	Low risk	Low risk	Low risk	Unclear risk	Unclear risk	Low risk
Chang et al. (34)	2020	Low risk	Unclear risk	Low risk	Low risk	Unclear risk	Unclear risk	Unclear risk
Gottlieb et al. (30)	2021	Low risk	Unclear risk	Low risk	Low risk	Unclear risk	Unclear risk	Low risk

### 3.4 The impact of TMS on FMA-UL in stroke patients

**Figure 2:**
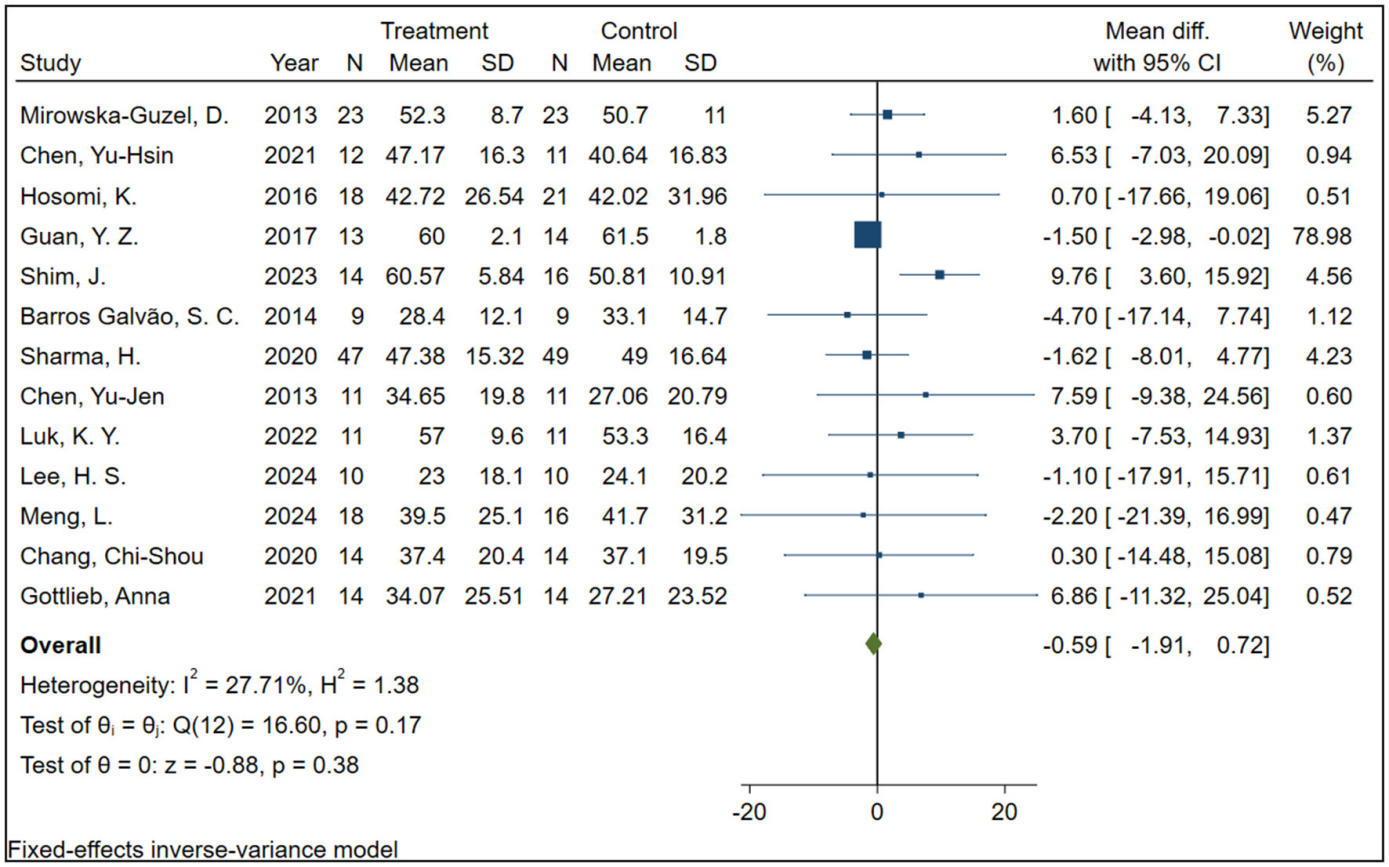
Forest plot of the effect of TMS on FMA-UL in stroke patients.

A total of 13 studies involving 433 participants investigated the effect of TMS on FMA-UL in stroke patients. Due to the relatively low heterogeneity of the studies (I^2^ = 27.71%, *P* = 0.17), a fixed-effects model was used for the analysis. The results indicated that, compared with the sham stimulation group, TMS did not have a statistically significant impact on FMA-UL in stroke patients (MD = −0.59, 95% CI: −1.91–0.72, *P* = 0.38) (Figure 2).

### 3.5 Secondary ending index gathered analysis

#### 3.5.1 The impact of TMS on FMA-LL in stroke patients

**Figure 3:**
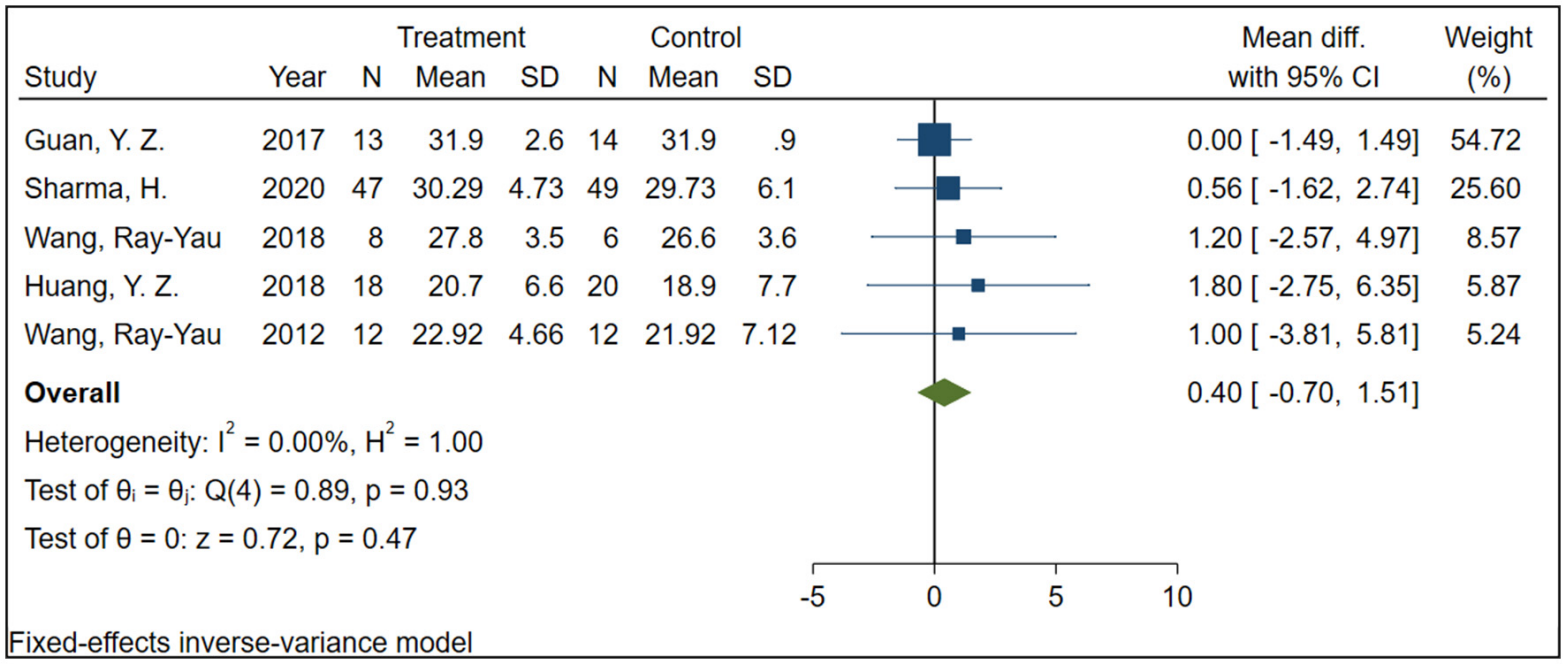
Forest plot of the effect of TMS on FMA-LL in stroke patients.

A total of 5 studies involving 199 participants investigated the effect of TMS on FMA-LL in stroke patients. Due to the low heterogeneity of the studies (I^2^ = 0.00%, *P* = 0.93), a fixed-effects model was used for the analysis. The results indicated that, compared with the sham stimulation group, TMS did not have a statistically significant impact on FMA-LL in stroke patients (MD = 0.40, 95% CI: −0.70–1.51, *P* = 0.47) (Figure 3).

#### 3.5.2 The impact of TMS on MAS in stroke patients

**Figure 4:**
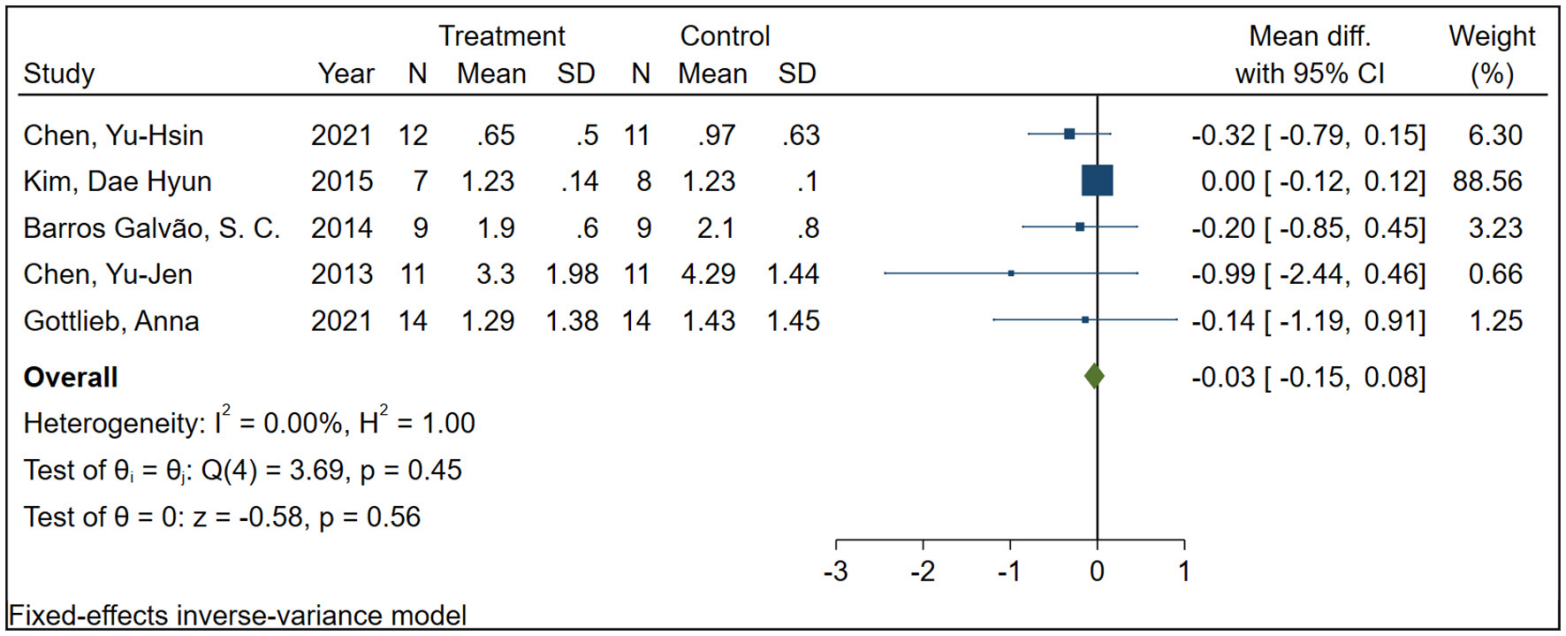
Forest plot of the effect of TMS on MAS in stroke patients.

A total of 5 studies involving 106 participants investigated the effect of TMS on the MAS in stroke patients. Due to the low heterogeneity of the studies (I^2^ = 0.00%, *P* = 0.45), a fixed-effects model was used for the analysis. The results indicated that, compared with the sham stimulation group, TMS did not have a statistically significant effect on MAS in stroke patients (MD = −0.03, 95% CI: −0.15–0.08, *P* = 0.56) (Figure 4).

#### 3.5.3 The impact of TMS on ARAT in stroke patients

**Figure 5:**
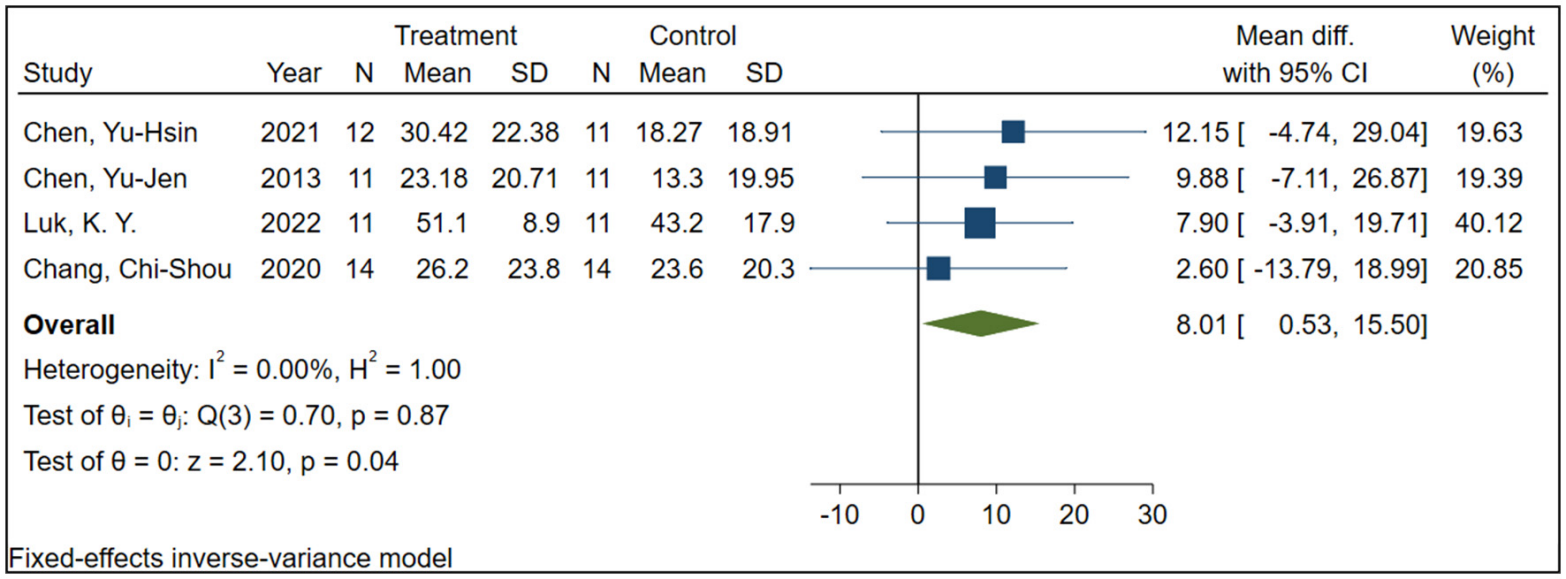
Forest plot of the effect of TMS on ARAT in stroke patients.

A total of 4 studies involving 95 participants investigated the effect of TMS on the ARAT in stroke patients. Due to the low heterogeneity of the studies (I^2^ = 0.00%, *P* = 0.87), a fixed-effects model was used for the analysis. The results indicated that, compared with the sham stimulation group, TMS significantly improved the ARAT in stroke patients (MD = 8.01, 95% CI: 0.53–15.50, *P* = 0.04) (Figure 5).

#### 3.5.4 The impact of TMS on BBT in stroke patients

**Figure 6:**
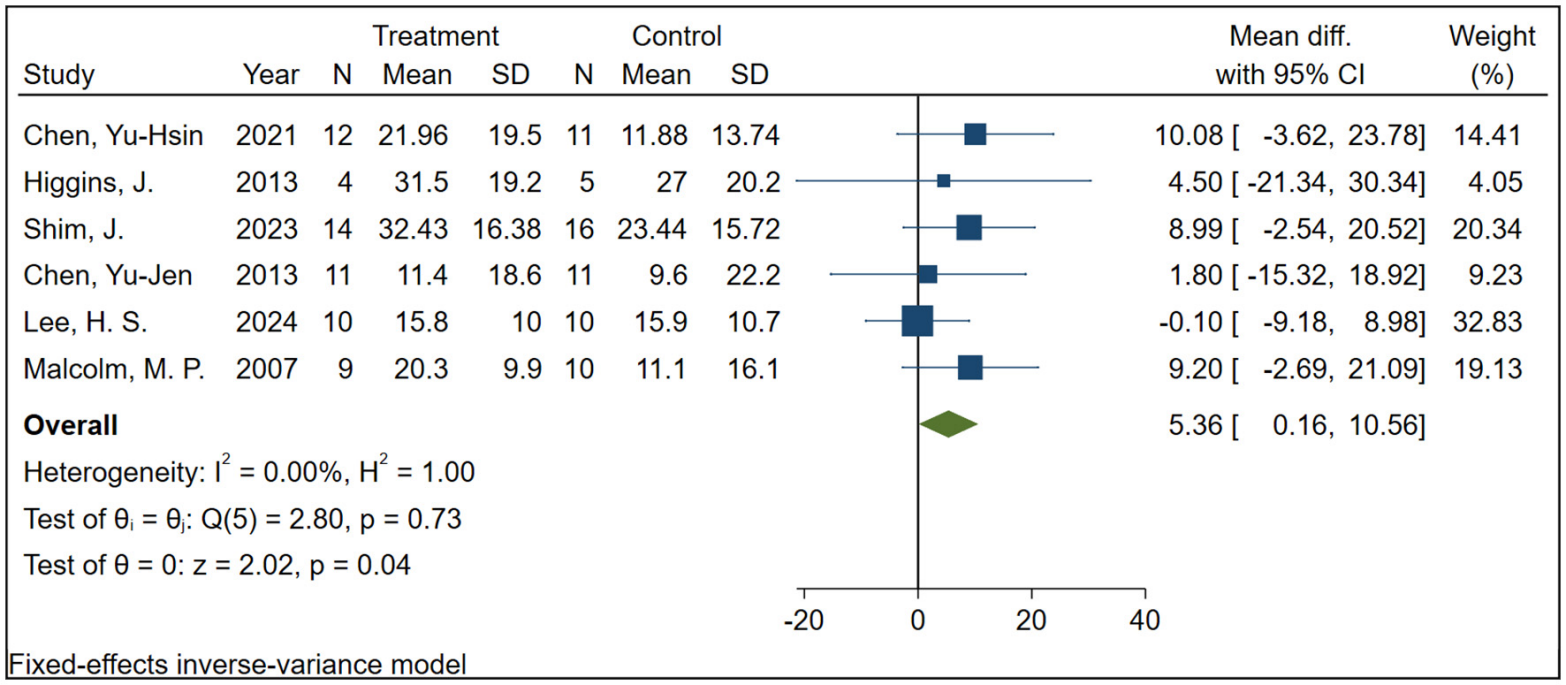
Forest plot of the effect of TMS on BBT in stroke patients.

A total of 6 studies involving 123 participants investigated the effect of TMS on the BBT in stroke patients. Due to the low heterogeneity of the studies (I^2^ = 0.00%, *P* = 0.73), a fixed-effects model was used for the analysis. The results indicated that, compared with the sham stimulation group, TMS significantly improved the BBT in stroke patients (MD = 5.36, 95% CI: 0.16–10.56, *P* = 0.04) (Figure 6).

#### 3.5.5 The impact of TMS on WMFT in stroke patients

**Figure 7:**
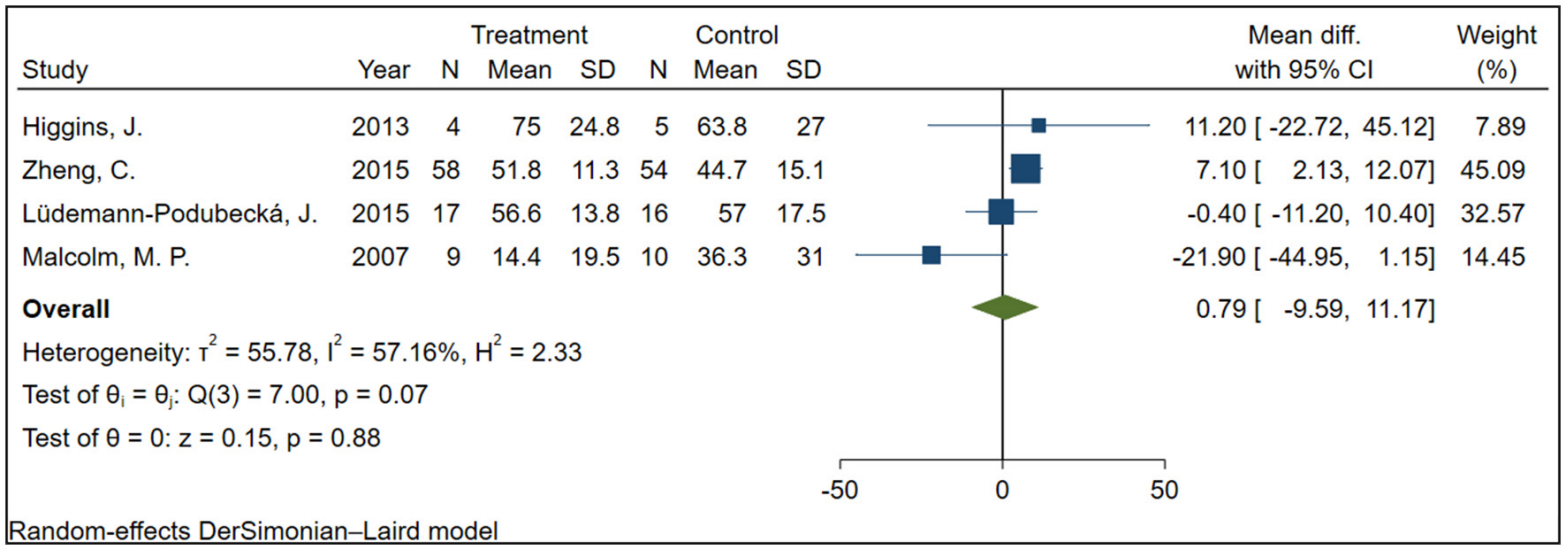
Forest plot of the effect of TMS on WMFT in stroke patients.

A total of 4 studies involving 173 participants investigated the effect of TMS on the WMFT in stroke patients. Due to the significant heterogeneity among the studies (I^2^ = 57.16%, *P* = 0.07), a random-effects model was used for the analysis. The results indicated that, compared with the sham stimulation group, TMS did not significantly affect the WMFT in stroke patients (MD = 0.79, 95% CI: −9.59–11.17, *P* = 0.88) (Figure 7).

### 3.6 Subgroup analysis

To explore the impact of TMS on FMA-UL, subgroup analysis were conducted based on intervention methods, cycles, and frequencies. The results showed that neither rTMS nor iTBS had a significant effect on FMA-UL (*P* > 0.05). The intervention cycle of weeks ≤ 2 did not significantly affect FMA-UL (MD = −1.36, 95% CI: −2.78–0.05, *P* = 0.059), whereas an intervention cycle of weeks > 2 significantly improved FMA-UL (MD = 4.44, 95% CI: 0.83–8.06, *P* = 0.016). However, low-frequency (≤ 1 Hz) and high-frequency (>1 Hz) stimulation did not significantly affect FMA-UL (*P* > 0.05) (Table 4).

**Table 4:** Subgroup analysis of the effect of TMS on FMA-UL in stroke patients.

Parameter	Group	Participants (E/C)	MD	95%CI	*P* (overall effect)	*P* (Heterogeneity)	I^2^, %
Type of intervention	rTMS	(164/169)	2.58	[−0.47, 5.62]	0.097	0.341	11.3
	iTBS	(50/50)	−1.32	[−2.78, 0.14]	0.076	0.485	0.0
	Total	(214/219)	−0.59	[−1.91, 0.72]	0.377	0.165	27.7
Intervention period	Week > 2	(69/70)	4.44	[0.83, 8.06]	0.016	0.200	33.2
	Week ≤ 2	(145/149)	−1.36	[−2.78, 0.05]	0.059	0.960	0.0
	Total	(214/219)	−0.59	[−1.91, 0.72]	0.377	0.165	27.7
Frequency	1 HZ	(104/106)	0.40	[−3.32, 4.12]	0.834	0.733	0.0
	>1 HZ	(110/113)	−0.74	[−2.14, 0.67]	0.306	0.047	51.0
	Total	(214/219)	−0.59	[−1.91, 0.72]	0.377	0.165	27.7

### 3.7 Sensitivity analysis

**Figure 8:**
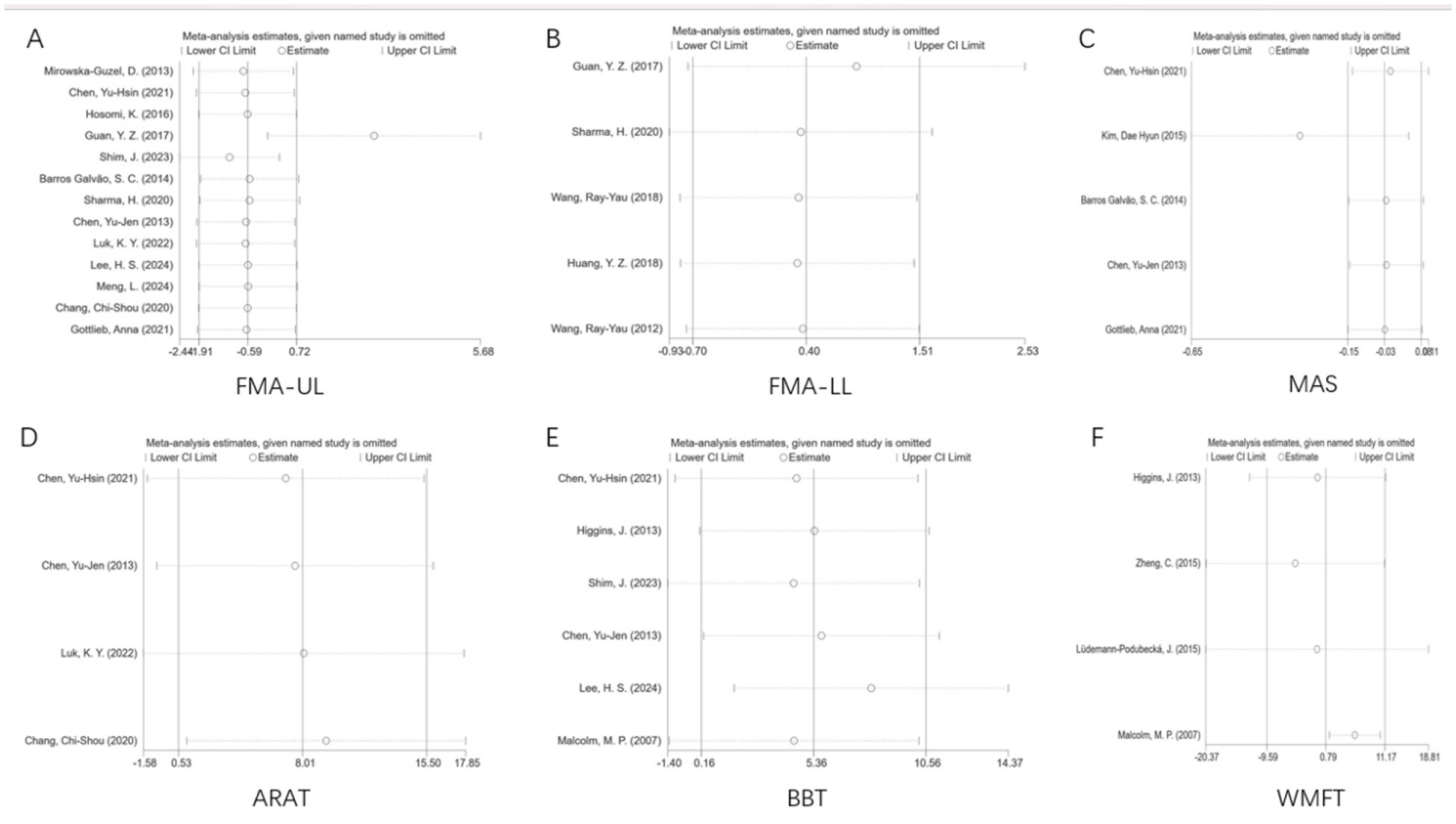
**(A)** FMA-UL, **(B)** FMA-LL, **(C)** MAS, **(D)** ARAT, **(E)** BBT, **(F)** WMFT. Chart of TMS sensitivity analysis for stroke patients with FMA-UL, FMA-LL, MAS, BBT, ARAT, and WMFT.

We performed sensitivity analysis using Stata 18.0. The results showed that for FMA-LL, ARAT, BBT, and WMFT, when the MD value from each randomized controlled trial was excluded, the MD values obtained from the random effect models did not exceed 95% CI of the total MD value. This indicates that the results of this study are relatively stable and robust. However, the results for FMA-UL and MAS may not be stable enough. Studies by Guan et al. (15) and Kim et al. (32), etc. had a greater impact on the overall results (Figure 8).

### 3.8 Publication bias detection

**Figure 9:**
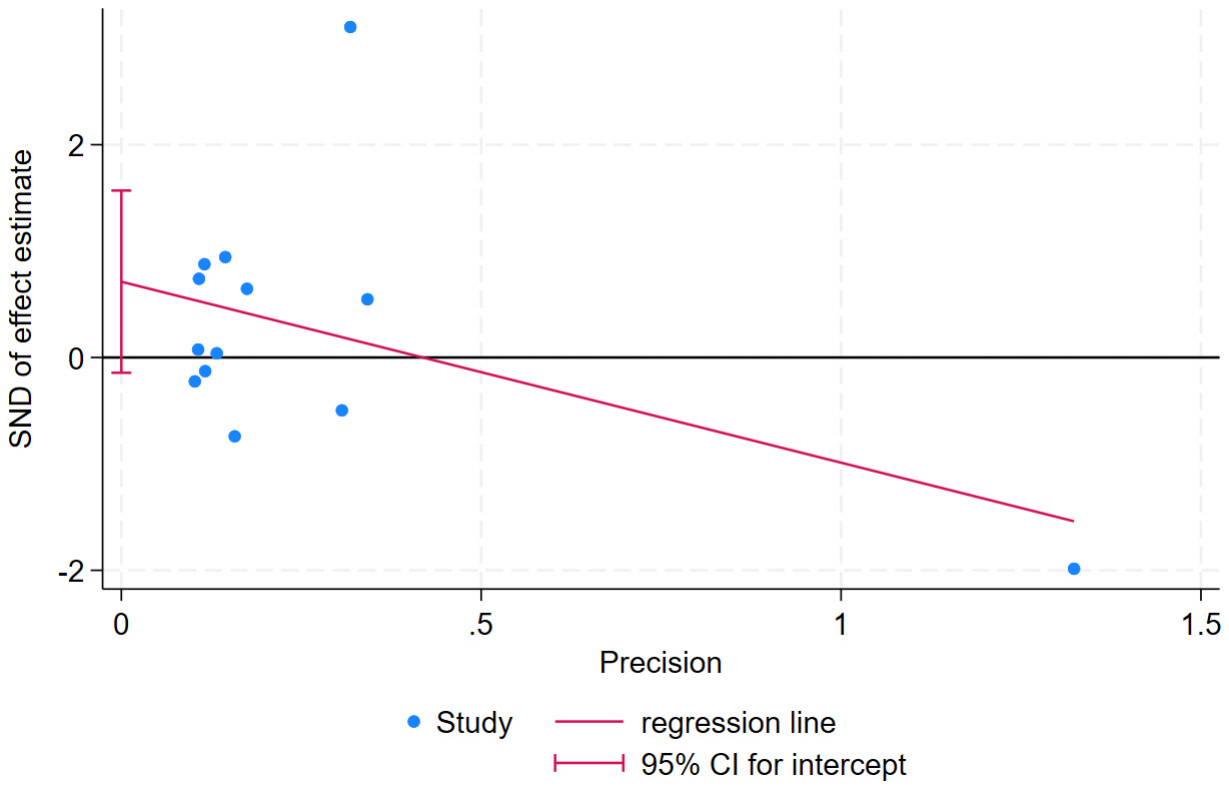
TMS publication bias for FMA-UL in stroke patients.

We performed a publication bias test using the Egger method for outcome indicators with more than 10 articles. The results showed that the publication bias for FMA-UL was not statistically significant (*P* = 0.094) (Figure 9).

## 4 Discussion

The present study evaluated the effects of TMS on several functional outcomes in patients with stroke, including FMA-UL, FMA-LL, MAS, ARAT, BBT, and WMFT. The pooled results indicated that rTMS was associated with significant improvements in ARAT and BBT scores (*P* = 0.04), suggesting potential benefits for upper-limb functional performance and manual dexterity. However, no statistically significant effects were observed for FMA-UL, FMA-LL, MAS, or WMFT outcomes (*P* > 0.05). Overall, statistical heterogeneity across the main outcomes was low, indicating that the direction of the effects was relatively consistent among the included studies. Nevertheless, low heterogeneity in statistical terms should not be interpreted as evidence of complete clinical or methodological homogeneity. Differences remained across studies in participant characteristics, including stroke subtype and baseline functional status (25, 33), as well as in intervention-related factors, such as stimulation modality, treatment duration, and stimulation frequency (21, 33). These variations may have contributed to differences in the observed therapeutic response to TMS. Furthermore, subgroup analyses were performed for FMA-UL according to stimulation modality, treatment duration, and stimulation frequency. The findings showed that rTMS, iTBS, low-frequency stimulation ≤ 1 Hz, high-frequency stimulation >1 Hz, and treatment duration ≤ 2 weeks were not associated with significant changes in FMA-UL scores (*P* > 0.05). In contrast, an intervention duration of more than 2 weeks was associated with a statistically significant improvement in FMA-UL scores (MD = 2.80, 95% CI: 0.06–5.54, *P* = 0.045), suggesting that the effects of TMS on upper-limb motor recovery may be partly dependent on treatment duration.

In the analysis of upper-limb outcomes, TMS was associated with significantly greater improvements in ARAT and BBT scores compared with sham stimulation. These findings suggest that TMS may have a favorable effect on upper-limb functional performance and manual dexterity in patients with stroke (31, 33). This observation is in line with the results reported by Chen et al. (35), further supporting the potential role of TMS in facilitating post-stroke upper-limb recovery. As a non-invasive neuromodulation technique, TMS has attracted increasing attention for its potential to promote motor rehabilitation after stroke. Its clinical benefits may extend beyond improvements in upper-limb dexterity and motor performance, potentially contributing to better activities of daily living and health-related quality of life (36). Mechanistically, TMS can influence cortical excitability in a frequency-dependent manner, exerting excitatory or inhibitory effects on motor cortical regions involved in movement control (37). In addition, TMS may facilitate neuroplastic reorganization, reflecting the capacity of the brain to adapt structurally and functionally in response to external stimulation (38). By modulating cortical excitability and promoting adaptive neural remodeling, TMS may contribute to neurological recovery and subsequent improvements in limb motor function (39, 40).

However, the pooled results did not demonstrate significant benefits of TMS over sham stimulation for FMA-UL, FMA-LL, MAS, or WMFT outcomes. The non-significant findings for FMA-UL, FMA-LL, and WMFT appear to differ from those reported by Qin et al. (41). Several explanations may account for this inconsistency. First, the stimulation dose or intensity used in the included studies may not have been sufficient to elicit robust neuroplastic adaptations, thereby attenuating the expected therapeutic response (13). Second, variations in participants' baseline motor function, stroke phase, and etiological characteristics may have contributed to heterogeneous responses to TMS (42). Third, stimulation frequency may be another important determinant of treatment efficacy, although the optimal frequency remains uncertain. For example, Zhang et al. (18) suggested that low-frequency TMS may enhance limb functional recovery after stroke, whereas Yang et al. (43) and Zhang et al. (44) reported more pronounced effects with high-frequency TMS. These inconsistent findings indicate that no universally accepted stimulation frequency has yet been established for TMS-based stroke rehabilitation, and that the optimal frequency may vary across individuals (45). Therefore, individualized treatment protocols should be considered in clinical practice to optimize therapeutic responses according to patient-specific characteristics. In contrast, the MAS results of the present study were consistent with those reported by Qin et al. (41). This finding may be explained by the fact that MAS primarily assesses changes in muscle tone, which are closely associated with alterations in corticospinal excitability. By modulating cortical excitability, TMS may influence descending motor pathways and subsequently affect spinal reflex circuits and γ-motor neuron activity, thereby contributing to short-term reductions in muscle spasticity (46, 47). Previous studies have also suggested that TMS may regulate abnormal reflex activity through excitatory or inhibitory modulation of the motor cortex, which could partly explain its potential role in improving post-stroke muscle tone abnormalities (48).

The subgroup analyses indicated that neither stimulation modality nor stimulation frequency was significantly associated with changes in FMA-UL scores (*P* > 0.05). Several factors may account for these non-significant findings. First, patients with stroke often differ substantially in clinical characteristics and underlying pathological conditions, which may lead to variable responses to TMS (42). Second, although both low- and high-frequency TMS protocols have been widely applied in post-stroke motor rehabilitation, current evidence does not consistently support the superiority of one frequency over another (12, 23). Third, TMS may influence motor recovery by regulating local neurotransmitter synthesis and transmission, as well as by modulating corticospinal tract excitability. Because different stimulation frequencies may engage these physiological processes through distinct pathways, their effects may vary across individuals, thereby reducing the likelihood of detecting a significant effect on FMA-UL scores (39). Fourth, the small number of eligible studies may have limited the statistical power of the subgroup analyses, which could partly explain the absence of significant effects across different TMS modalities and frequency categories. Notably, treatment duration appeared to be an important factor influencing the therapeutic response. An intervention period of more than 2 weeks was associated with a significant improvement in FMA-UL scores, whereas interventions lasting 2 weeks or less showed no significant effect. This finding suggests that the benefits of TMS may be time-dependent, as sustained and repeated stimulation may be required to induce cumulative neuroplastic changes, whereas short-term interventions may be insufficient to produce stable functional recovery (49). From a clinical perspective, this result suggests that extending the treatment duration may be more relevant to optimizing therapeutic efficacy than simply modifying stimulation frequency or modality. Moreover, given the heterogeneity in individual responses to stimulation parameters, clinical application of TMS should emphasize personalized treatment strategies. In particular, stimulation protocols should be tailored according to patient-specific factors, such as stroke phase and severity of functional impairment, rather than relying on a single standardized parameter setting (50).

Although several subgroup analyses were conducted in the present study, adjustments for multiple comparisons were not performed. This methodological limitation may increase the probability of type I error and false-positive findings. Therefore, the statistically significant result observed for interventions lasting more than 2 weeks should be interpreted cautiously. Future research should consider applying appropriate correction procedures, such as the Bonferroni method or false discovery rate adjustment, to control for multiple testing and enhance the reliability of subgroup findings (51). In addition, larger and adequately powered studies are warranted to confirm whether treatment duration independently influences the efficacy of TMS in post-stroke limb functional recovery.

In addition, although risk of bias was evaluated using the Cochrane assessment tool, several included studies were judged to have unclear risks in relation to allocation concealment and blinding procedures. These methodological concerns may have introduced potential selection bias, performance bias, and detection bias. This issue is particularly relevant for outcomes that involve a degree of assessor judgment, such as the FMA and WMFT, where inadequate blinding may compromise the accuracy and objectivity of outcome assessment. Consequently, the internal validity of the present findings should be interpreted with caution.

All studies included in this review were randomized controlled trials, and the baseline characteristics of participants were generally balanced between groups, which strengthens the credibility of the findings to some extent. Nevertheless, several limitations should be considered when interpreting the results. First, the number of eligible studies was relatively small, and some outcomes were based on limited sample sizes. This may have reduced the statistical power of the analyses and increased the uncertainty of the pooled estimates. Second, variations existed across studies in intervention protocols, including stimulation modality, stimulation frequency, and treatment duration. Such methodological and clinical differences may have contributed to variability in treatment effects and may limit the generalizability of the findings. Third, the outcome measures used in the included studies, such as the FMA, MAS, ARAT, BBT, and WMFT, assess different aspects of motor function and differ in their sensitivity to clinical change. This heterogeneity in outcome assessment may further complicate the interpretation of the results. Finally, the present study did not fully account for potential effect modifiers such as patient age and stimulation target, both of which may influence individual responses to TMS. Moreover, the molecular mechanisms underlying TMS-induced recovery after stroke remain incompletely understood, limiting a more detailed mechanistic interpretation of the observed effects. Future large-scale, well-designed randomized controlled trials are therefore warranted to confirm these findings, optimize stimulation protocols, and further clarify the biological mechanisms through which TMS may improve limb function in patients with stroke.

## 5 Conclusion

TMS has a positive effect on the limb function of stroke patients, particularly in improving the flexibility and movement function of the upper limbs. The subgroup analysis indicated that the FMA-UL score is closely related to the duration of the intervention cycle, while the intervention method and frequency have less impact. However, due to the limited number of studies, further research is necessary to explore the full potential of TMS in enhancing limb function in stroke patients.

## Data Availability

The original contributions presented in the study are included in the article/Supplementary material, further inquiries can be directed to the corresponding author.

## References

[1] GBD 2016 Stroke Collaborators. Global, regional, and national burden of stroke, 1990–2016: a systematic analysis for the global burden of disease study 2016. Lancet Neurol. (2019) 18:439–58. doi: 10.1016/S1474-4422(19)30034-1 30871944 PMC6494974

[2] GBD 2019 Stroke Collaborators. Global, regional, and national burden of stroke and its risk factors, 1990–2019: a systematic analysis for the Global burden of disease study 2019. Lancet Neurol. (2021) 20:795–820. doi: 10.1016/S1474-4422(21)00252-0 34487721 PMC8443449

[3] Wang J Wu Z Hong S Ye H Zhang Y Lin Q . Cerebellar transcranial magnetic stimulation for improving balance capacity and activity of daily living in stroke patients: a systematic review and meta-analysis. BMC Neurol. (2024) 24:205. doi: 10.1186/s12883-024-03720-1 38879485 PMC11179288

[4] Winstein CJ Stein J Arena R Bates B Cherney LR Cramer SC . Guidelines for adult stroke rehabilitation and recovery: a guideline for healthcare professionals from the American heart association/American stroke association. Stroke. (2016) 47:e98–169. doi: 10.1161/STR.0000000000000098 27145936

[5] Huang J Ji JR Liang C Zhang YZ Sun HC Yan YH . Effects of physical therapy-based rehabilitation on recovery of upper limb motor function after stroke in adults: a systematic review and meta-analysis of randomized controlled trials. Ann Palliat Med. (2022) 11:521–31. doi: 10.21037/apm-21-3710 35249330

[6] Kakuda W Abo M Kobayashi K Momosaki R Yokoi A Fukuda A . Application of combined 6-Hz primed low-frequency rTMS and intensive occupational therapy for upper limb hemiparesis after stroke. NeuroRehabilitation. (2011) 29:365–71. doi: 10.3233/NRE-2011-0714 22207064

[7] Simonetta-Moreau M. Non-invasive brain stimulation (NIBS) and motor recovery after stroke. Ann Phys Rehabil Med. (2014) 57:530–42. doi: 10.1016/j.rehab.2014.08.003 25193774

[8] Edwardson MA Lucas TH Carey JR Fetz EE. New modalities of brain stimulation for stroke rehabilitation. Exp Brain Res. (2013) 224:335–58. doi: 10.1007/s00221-012-3315-1 23192336 PMC4438996

[9] Hsu WY Cheng CH Liao KK Lee IH Lin YY. Effects of repetitive transcranial magnetic stimulation on motor functions in patients with stroke: a meta-analysis. Stroke. (2012) 43:1849–57. doi: 10.1161/STROKEAHA.111.649756 22713491

[10] Li R Liu S Li T Yang K Wang X Wang W. The stratified effects of repetitive transcranial magnetic stimulation in upper limb motor impairment recovery after stroke: a meta-analysis. Front Neurol. (2024) 15:1369836. doi: 10.3389/fneur.2024.1369836 38628695 PMC11020108

[11] Keesukphan A Suntipap M Thadanipon K Boonmanunt S Numthavaj P McKay GJ . Effects of electrical and magnetic stimulation on upper extremity function after stroke: a systematic review and network meta-analysis. PM R. (2025) 17:978–93. doi: 10.1002/pmrj.13356 40396624 PMC12345400

[12] Shim J Lee S. Effects of high-frequency repetitive transcranial magnetic stimulation combined with motor learning on motor function and grip force of the upper limbs and activities of daily living in patients with a subacute stroke. Int J Environ Res Public Health. (2023) 20:6093. doi: 10.3390/ijerph20126093 37372680 PMC10297839

[13] Malcolm MP Triggs WJ Light KE Gonzalez Rothi LJ Wu S Reid K . Repetitive transcranial magnetic stimulation as an adjunct to constraint-induced therapy: an exploratory randomized controlled trial. Am J Phys Med Rehabil. (2007) 86:707–15. doi: 10.1097/PHM.0b013e31813e0de0 17709994 PMC2605430

[14] Hosomi K Morris S Sakamoto T Taguchi J Maruo T Kageyama Y . Daily repetitive transcranial magnetic stimulation for poststroke upper limb paresis in the subacute period. J Stroke Cerebrovasc Dis. (2016) 25:1655–64. doi: 10.1016/j.jstrokecerebrovasdis.2016.02.024 27067882

[15] Guan YZ Li J Zhang XW Wu S Du H Cui LY . Effectiveness of repetitive transcranial magnetic stimulation (rTMS) after acute stroke: a one-year longitudinal randomized trial. CNS Neurosci Ther. (2017) 23:940–6. doi: 10.1111/cns.12762 28971620 PMC6492666

[16] Sharma H Vishnu VY Kumar N Sreenivas V Rajeswari MR Bhatia R . Efficacy of low-frequency repetitive transcranial magnetic stimulation in ischemic stroke: a double-blind randomized controlled trial. Arch Rehabil Res Clin Transl. (2020) 2:100039. doi: 10.1016/j.arrct.2020.100039 33543068 PMC7853333

[17] Meng L Ge Y Tsang RCC Zhang W Liu X Li S . rTMS for poststroke pusher syndrome: a randomized, patient-blinded controlled clinical trial. Neurorehabil Neural Repair. (2024) 38:670–9. doi: 10.1177/15459683241268537 39104197

[18] Zhang L Xing G Shuai S Guo Z Chen H McClure MA . Low-frequency repetitive transcranial magnetic stimulation for stroke-induced upper limb motor deficit: a meta-analysis. Neural Plast. (2017) 2017:2758097. doi: 10.1155/2017/2758097 29435371 PMC5756908

[19] Wang R-Y Wang F-Y Huang S-F Yang Y-R. High-frequency repetitive transcranial magnetic stimulation enhanced treadmill training effects on gait performance in individuals with chronic stroke: a double-blinded randomized controlled pilot trial. Gait Posture. (2019) 68:382–7. doi: 10.1016/j.gaitpost.2018.12.023 30586670

[20] Xi X Wang H Han L Ding M Li J Qiao C . Meta-analysis of repetitive transcranial magnetic stimulation combined with task-oriented training on upper limb function in stroke patients with hemiplegia. Medicine. (2023) 102:e33771. doi: 10.1097/MD.0000000000033771 37266626 PMC10238008

[21] Mirowska-Guzel D Gromadzka G Seniow J Lesniak M Bilik M Waldowski K . Association between BDNF-196 G>A and BDNF-270 C>T polymorphisms, BDNF concentration, and rTMS-supported long-term rehabilitation outcome after ischemic stroke. NeuroRehabilitation. (2013) 32:573–82. doi: 10.3233/NRE-130879 23648611

[22] Higgins J Koski L Xie H. Combining rTMS and task-oriented training in the rehabilitation of the arm after stroke: a pilot randomized controlled trial. Stroke Res Treat. (2013) 2013:539146. doi: 10.1155/2013/539146 24363954 PMC3865731

[23] Zheng C Liao W Xia W. Effect of combined low-frequency repetitive transcranial magnetic stimulation and virtual reality training on upper limb function in subacute stroke: a double-blind randomized controlled trail. J Huazhong Univ Sci Technol Med Sci. (2015) 35:248–54. doi: 10.1007/s11596-015-1419-0 25877360

[24] Lüdemann-Podubecká J Bösl K Theilig S Wiederer R Nowak DA. The effectiveness of 1 Hz rTMS over the primary motor area of the unaffected hemisphere to improve hand function after stroke depends on hemispheric dominance. Brain Stimul. (2015) 8:823–30. doi: 10.1016/j.brs.2015.02.004 25828427

[25] Barros Galvão SC Borba Costa dos Santos R Borba dos Santos P Cabral ME Monte-Silva K. Efficacy of coupling repetitive transcranial magnetic stimulation and physical therapy to reduce upper-limb spasticity in patients with stroke: a randomized controlled trial. Arch Phys Med Rehabil. (2014) 95:222–9. doi: 10.1016/j.apmr.2013.10.023 24239881

[26] Luk KY Ouyang HX Pang MYC. Low-frequency rTMS over contralesional M1 increases ipsilesional cortical excitability and motor function with decreased interhemispheric asymmetry in subacute stroke: a randomized controlled study. Neural Plast. (2022) 2022:3815357. doi: 10.1155/2022/3815357 35035473 PMC8756161

[27] Lee HS Kim S Kim H Baik SM Kim DH Chang WH. No additional effects of sequential facilitatory cerebral and cerebellar rTMS in subacute stroke patients. J Pers Med. (2024) 14:687. doi: 10.3390/jpm14070687 39063941 PMC11278256

[28] Huang YZ Lin LF Chang KH Hu CJ Liou TH Lin YN. Priming with 1-Hz repetitive transcranial magnetic stimulation over contralesional leg motor cortex does not increase the rate of regaining ambulation within 3 months of stroke: a randomized controlled trial. Am J Phys Med Rehabil. (2018) 97:339–45. doi: 10.1097/PHM.0000000000000850 29023249

[29] Wang R-Y Tseng H-Y Liao K-K Wang C-J Lai K-L Yang Y-R. rTMS combined with task-oriented training to improve symmetry of interhemispheric corticomotor excitability and gait performance after stroke: a randomized trial. Neurorehabil Neural Repair. (2012) 26:222–30. doi: 10.1177/1545968311423265 21974983

[30] Gottlieb A Boltzmann M Schmidt SB Gutenbrunner C Krauss JK Stangel M . Treatment of upper limb spasticity with inhibitory repetitive transcranial magnetic stimulation: a randomized placebo-controlled trial. NeuroRehabilitation. (2021) 49:425–34. doi: 10.3233/NRE-210088 34542038

[31] Chen Y-H Chen C-L Huang Y-Z Chen H-C Chen C-Y Wu C-Y . Augmented efficacy of intermittent theta burst stimulation on the virtual reality-based cycling training for upper limb function in patients with stroke: a double-blinded, randomized controlled trial. J Neuroeng Rehabil. (2021) 18:91. doi: 10.1186/s12984-021-00885-5 34059090 PMC8166006

[32] Kim DH Shin JC Jung S Jung T-M Kim DY. Effects of intermittent theta burst stimulation on spasticity after stroke. Neuroreport. (2015) 26:561–6. doi: 10.1097/WNR.0000000000000388 26011507 PMC4498651

[33] Chen Y-J Huang Y-Z Chen C-Y Chen C-L Chen H-C Wu C-Y . Intermittent theta burst stimulation enhances upper limb motor function in patients with chronic stroke: a pilot randomized controlled trial. BMC Neurol. (2019) 19:69. doi: 10.1186/s12883-019-1302-x 31023258 PMC6485156

[34] Chang C-S Chen C-L Chen R-S Chen H-C Chen C-Y Chung C-Y . Synergistic efficacy of repetitive peripheral magnetic stimulation on central intermittent theta burst stimulation for upper limb function in patients with stroke: a double-blinded, randomized controlled trial. J Neuroeng Rehabil. (2024) 21:49. doi: 10.1186/s12984-024-01341-w 38589875 PMC11000298

[35] Chen G Lin T Wu M Cai G Ding Q Xu J . Effects of repetitive transcranial magnetic stimulation on upper-limb and finger function in stroke patients: a systematic review and meta-analysis of randomized controlled trials. Front Neurol. (2022) 13:940467. doi: 10.3389/fneur.2022.940467 35968309 PMC9372362

[36] Motamed Vaziri P Bahrpeyma F Firoozabadi M Forough B Hatef B Sheikhhoseini R . Low frequency repetitive transcranial magnetic stimulation to improve motor function and grip force of upper limbs of patients with hemiplegia. Iran Red Crescent Med J. (2014) 16:e13579. doi: 10.5812/ircmj.13579 25389476 PMC4222002

[37] Cavaleri R Schabrun SM Chipchase LS. The number of stimuli required to reliably assess corticomotor excitability and primary motor cortical representations using transcranial magnetic stimulation (TMS): a systematic review and meta-analysis. Syst Rev. (2017) 6:48. doi: 10.1186/s13643-017-0440-8 28264713 PMC5340029

[38] Bhandari A Radhu N Farzan F Mulsant BH Rajji TK Daskalakis ZJ . A meta-analysis of the effects of aging on motor cortex neurophysiology assessed by transcranial magnetic stimulation. Clin Neurophysiol. (2016) 127:2834–45. doi: 10.1016/j.clinph.2016.05.363 27417060 PMC4956500

[39] Bai Z Zhang J Fong KNK. Effects of transcranial magnetic stimulation in modulating cortical excitability in patients with stroke: a systematic review and meta-analysis. J Neuroeng Rehabil. (2022) 19:24. doi: 10.1186/s12984-022-00999-4 35193624 PMC8862292

[40] McDonnell MN Stinear CM. TMS measures of motor cortex function after stroke: a meta-analysis. Brain Stimul. (2017) 10:721–34. doi: 10.1016/j.brs.2017.03.008 28385535

[41] Qin J Hong Z Wang J Zhang Y Zhuang H Hong S . Effectiveness of dual-site transcranial magnetic stimulation on motor function and activities of daily living in stroke patients: a systematic review and meta-analysis of randomized controlled trials. Front Neurol. (2025) 16:1630876. doi: 10.3389/fneur.2025.1630876 40726624 PMC12301190

[42] Kakuda W Abo M Shimizu M Sasanuma J Okamoto T Yokoi A . A multi-center study on low-frequency rTMS combined with intensive occupational therapy for upper limb hemiparesis in post-stroke patients. J Neuroeng Rehabil. (2012) 9:4. doi: 10.1186/1743-0003-9-4 22264239 PMC3271959

[43] Yang Y Chang W Ding J Xu H Wu X Ma L . Effects of different modalities of transcranial magnetic stimulation on post-stroke cognitive impairment: a network meta-analysis. Neurol Sci. (2024) 45:4399–416. doi: 10.1007/s10072-024-07504-w 38600332

[44] Zhang Q Qiu Z. Therapeutic effects of repetitive transcranial magnetic stimulation in patients with cerebral palsy: a systematic review and network meta-analysis. Neurol Sci. (2024) 45:1953–67. doi: 10.1007/s10072-023-07235-4 38117402

[45] Xiang H Sun J Tang X Zeng K Wu X. The effect and optimal parameters of repetitive transcranial magnetic stimulation on motor recovery in stroke patients: a systematic review and meta-analysis of randomized controlled trials. Clin Rehabil. (2019) 33:847–64. doi: 10.1177/0269215519829897 30773896

[46] Di Lazzaro V Ziemann U Lemon RN. State of the art: physiology of transcranial motor cortex stimulation. Brain Stimul. (2008) 1:345–62. doi: 10.1016/j.brs.2008.07.004 20633393

[47] Hallett M. Transcranial magnetic stimulation: a primer. Neuron. (2007) 55:187–99. doi: 10.1016/j.neuron.2007.06.026 17640522

[48] Lefaucheur JP André-Obadia N Antal A Ayache SS Baeken C Benninger DH . Evidence-based guidelines on the therapeutic use of repetitive transcranial magnetic stimulation (rTMS). Clin Neurophysiol. (2014) 125:2150–206. doi: 10.1016/j.clinph.2014.05.021 25034472

[49] Cramer SC Sur M Dobkin BH O'Brien C Sanger TD Trojanowski JQ . Harnessing neuroplasticity for clinical applications. Brain. (2011) 134:1591–609. doi: 10.1093/brain/awr039 21482550 PMC3102236

[50] Ward NS. Restoring brain function after stroke - bridging the gap between animals and humans. Nat Rev Neurol. (2017) 13:244–55. doi: 10.1038/nrneurol.2017.34 28303914

[51] Bender R Lange S. Adjusting for multiple testing–when and how? J Clin Epidemiol. (2001) 54:343–9. doi: 10.1016/S0895-4356(00)00314-0 11297884

